# Design and Construction of Artificial Biological Systems for One-Carbon Utilization

**DOI:** 10.34133/bdr.0021

**Published:** 2023-10-31

**Authors:** Wei Zhong, Hailong Li, Yajie Wang

**Affiliations:** ^1^ Westlake Center of Synthetic Biology and Integrated Bioengineering, School of Engineering, Westlake University, Hangzhou 310000, PR China.; ^2^ School of Materials Science and Engineering, Zhejiang University, Zhejiang Province, Hangzhou 310000, PR China.

## Abstract

The third-generation (3G) biorefinery aims to use microbial cell factories or enzymatic systems to synthesize value-added chemicals from one-carbon (C1) sources, such as CO_2_, formate, and methanol, fueled by renewable energies like light and electricity. This promising technology represents an important step toward sustainable development, which can help address some of the most pressing environmental challenges faced by modern society. However, to establish processes competitive with the petroleum industry, it is crucial to determine the most viable pathways for C1 utilization and productivity and yield of the target products. In this review, we discuss the progresses that have been made in constructing artificial biological systems for 3G biorefineries in the last 10 years. Specifically, we highlight the representative works on the engineering of artificial autotrophic microorganisms, tandem enzymatic systems, and chemo-bio hybrid systems for C1 utilization. We also prospect the revolutionary impact of these developments on biotechnology. By harnessing the power of 3G biorefinery, scientists are establishing a new frontier that could potentially revolutionize our approach to industrial production and pave the way for a more sustainable future.

## Introduction

The trend of rising atmospheric carbon dioxide (CO_2_) concentrations remains unabated. The projections indicate a potential atmospheric CO_2_ concentration of 500 ppm by the year 2045 [1], which will cause tremendous ecological and environmental problems, including global warming, extreme weather events, rising sea levels, and wildlife extinction [2,3]. The world’s heavy dependency on fossil fuels and accelerated energy consumption majorly contribute to greenhouse gas emissions and global warming [4]. Biotechnologies provide eco-friendly alternatives for the production of fuels and chemicals. The biorefineries using sugar-based feedstocks [the first generation (1G)] and biomass feedstocks [the second generation (2G)] are developed to produce biofuels and value-added chemicals in a carbon-neutral manner [5]. Although 1G and 2G biorefineries are capable of producing biofuels and value-added compounds from various feedstocks, including food crops like sugar beet and sugar cane, energy crops like lignocellulosic masses, and waste materials such as the organic fraction of municipal solid waste or landfill leachate, they rarely assimilate C1 sources into either biomass or other value-added products [6]. Recently, there is increasing interests to construct microbes or tandem enzymatic reactions for CO_2_ fixation and utilization.

3G biorefinery aims to use engineered enzymes or microbes to produce value-added compounds from one-carbon (C1) sources such as formaldehyde, formic acid, methanol, methane, and CO_2_, with the energy supplies from the chemical bond breaking, electricity, and light [7]. Although microbes with native C1 assimilation machinery have been reported to have a great potential to produce valuable compounds from C1 sources [8–11], some of them are not applicable in scale-up biomanufacturing processes because of low carbon fixation efficiency and narrow product scope [12–15]. Differently, heterotrophic microorganisms, such as *Escherichia coli* and yeasts, such as *Pichia pastoris*, *Saccharomyces cerevisiae*, and *Ogataea polymorpha*, have been facilitated with well-developed synthetic biology tools and engineered to produce a wide range of biofuels, fine chemicals, and natural products [16–18]. Installing C1 fixation machinery in these heterotrophic microbes could potentially be a significant strategy to enhance the low carbon fixation efficiency and poor productivity of targeted compounds. Computational biology offers a viable way for designing and constructing artificial C1 fixation pathways in vitro, which can further enhance the efficiency of C1 fixation and production yield [19–25]. Additionally, since C1 assimilation is an energy-consuming process, constructing chemo-bio hybrid systems to drive the assimilation process with electricity and light has emerged as a popular strategy. In this review, we discuss the representative artificial biological systems developed within the recent 10 years for C1 fixation, focusing on engineered heterotrophs for C1 assimilation, in vitro enzymatic cascades for CO_2_ fixation, and electrical-to-chemical and solar-to-chemical conversion for C1 utilization.

## Engineering Artificial Biological Systems for C1 Utilization

**Fig. 1.**
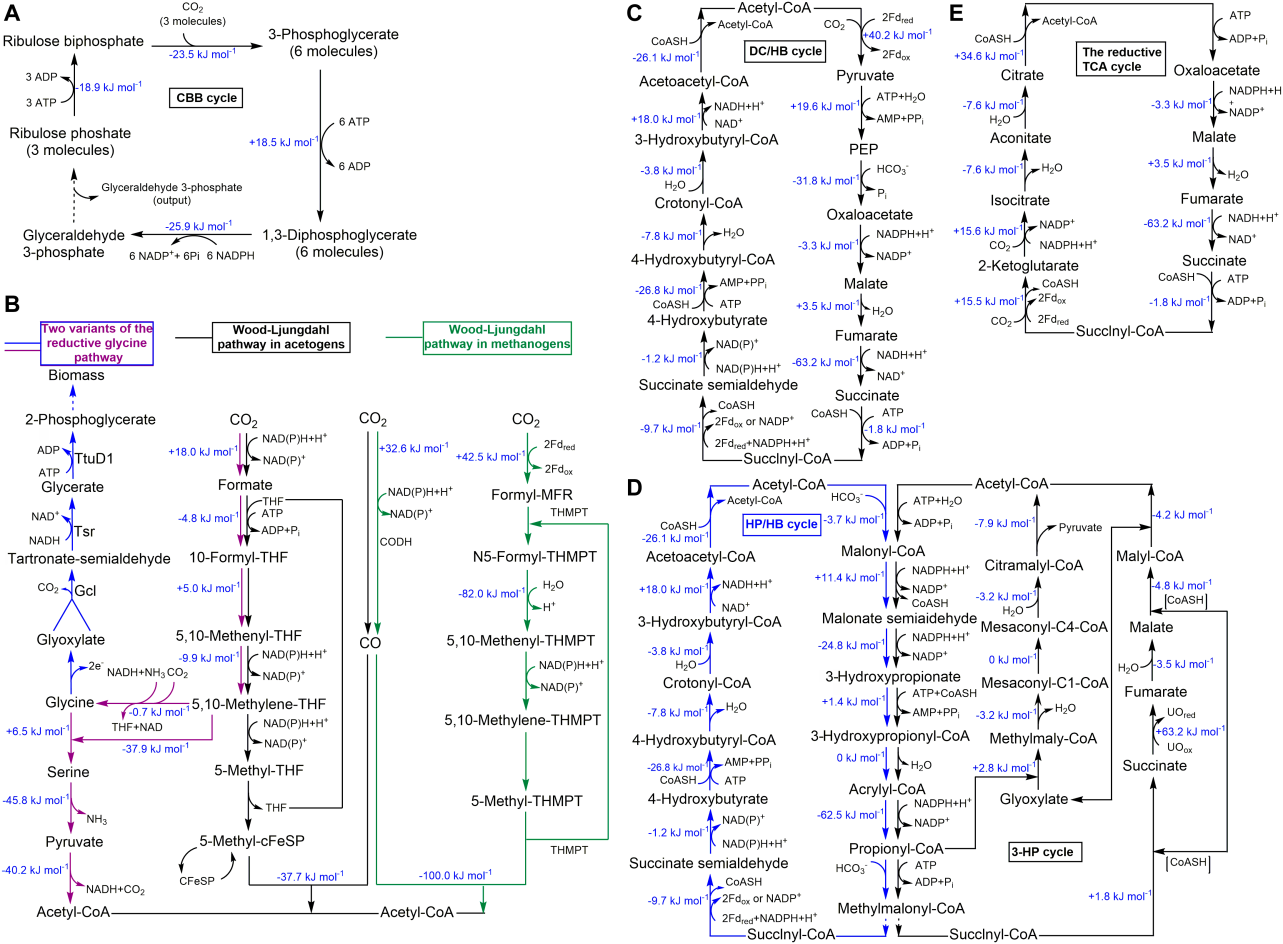
Existing naturally existed CO_2_ fixation pathways. (A) CBB cycle. This cycle is closely related to the pentose phosphate pathway [26]. (B) Reductive glycine pathway (in purple and blue) [27] and Wood–Ljungdahl pathway in acetogens (in black) and methanolgens (in green) [30]. (C) DC/HB cycle. This cycle fixes 1 mol of CO_2_ by pyruvate synthase and 1 mol of bicarbonate by phosphoenolpyruvate (PEP) carboxylase [28]. (D) HP/HB cycle (in blue) and 3-HP cycle (in black). These cycles assimilate 2 mol of bicarbonate by acetyl-CoA/propionyl-CoA carboxylase [29]. (E) Reductive TCA cycle. This cycle fixes 2 mol of CO_2_ by reversing the oxidative TCA cycle [31]. The changes in the Gibbs energy calculated by eQuilibrator [183,184] are shown in blue.

**Fig. 2.**
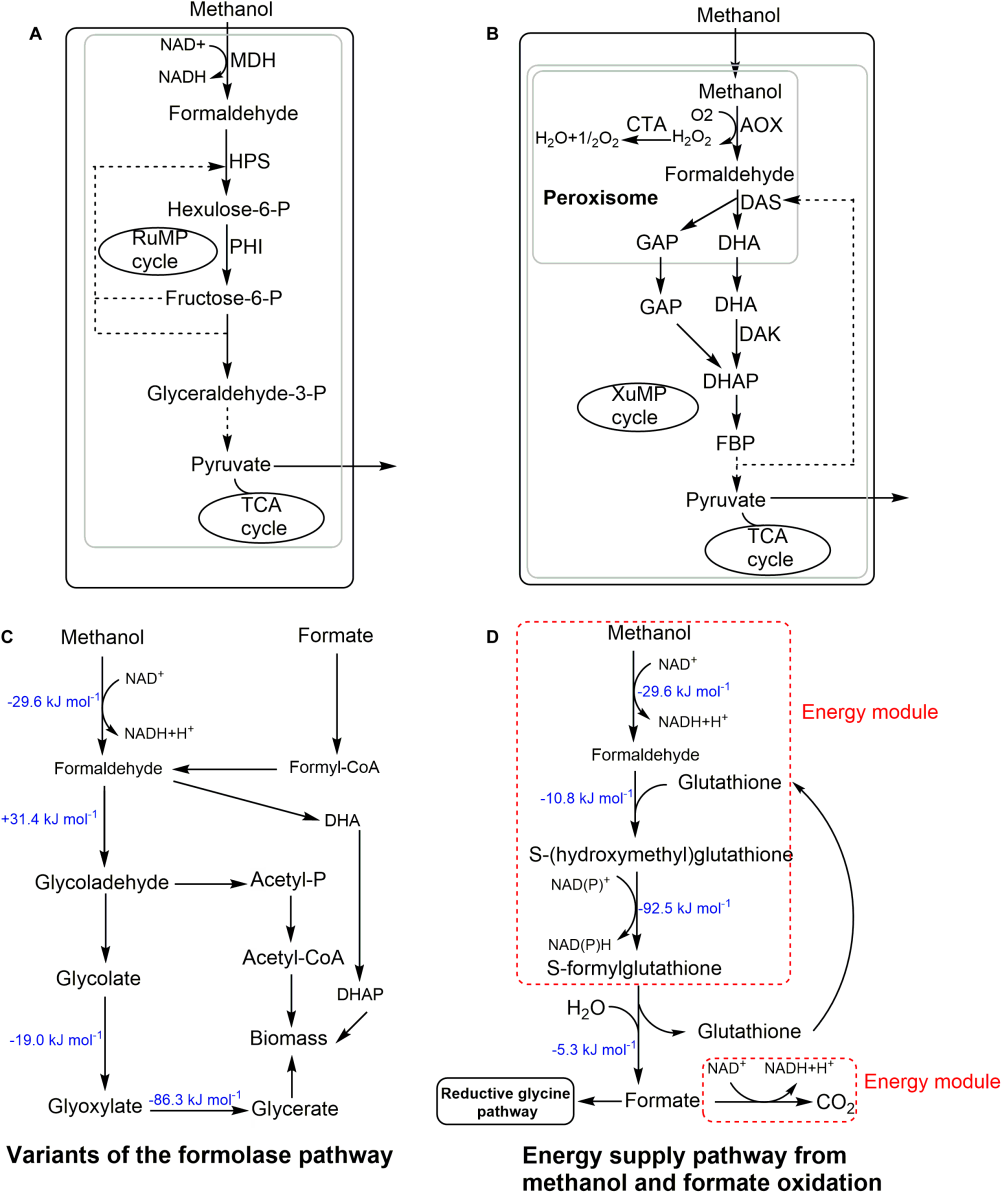
The methanol and formate assimilation pathways. (A) Methanol is assimilated through the RuMP cycle [32]. (B) Methanol is assimilated through the XuMP cycle [32]. (C) Methanol and formate are assimilated through the formolase pathway [33]. (D) Energy supply pathway: methanol oxidation and formate oxidation [24]. The changes in the Gibbs energy calculated by the eQuilibrator [183,184] are shown in blue.

The natural CO_2_ fixation pathways discovered so far include the Calvin–Benson–Bassham (CBB) cycle [26], the reductive glycine pathway [27], the dicarboxylate/4-hydroxybutyrate (DC/HB) cycle [28], the 3-hydroxypropionate/4-hydroxybutyrate (HP/HB) cycle [29], the Wood–Lindahl pathway [30], and the reductive tricarboxylic acid cycle (TCA cycle) [31] (Fig. 1). In some cases, formate is converted to pyruvate through the reductive glycine (rGly) pathway, which consists of 3 modules: formate to 5,10-methylene-THF (tetrahydrofolate), glycine cleavage systems (GCSs), and glycine to pyruvate [24] (Fig.1). Methanol is first converted to formaldehyde and then assimilated through the native pathways, such as the ribulose monophosphate cycle (RuMP cycle), the CBB cycle, the serine cycle, or the xylulose monophosphate cycle (XuMP cycle) [32] (Fig. 2A and B). However, formate and methanol can also be directly assimilated into the reductive acetyl-CoA (coenzyme A) pathway through acetogen strains and variants of the formolase pathway [33] (Fig. 2C). On the other hand, methene is oxidized to methanol by methane monooxygenase and then assimilated through the methanol oxidation pathway [34]. Carbon monoxide (CO) can be used as a source of carbon or energy for growth in some microorganisms under aerobic and anaerobic conditions [35]. In the presence of CO dehydrogenase, CO can be converted to CO_2_, hydrogen sulfide, hydrogen, acetate, and methane, which are associated with a range of respiratory pathways, including but not limited to desulfurication, hydrogenogenesis, acetogenesis, and methanogenesis [35].

Artificial biological systems for C1 fixation are either reconstructed based on the native one or de novo designed. This section will highlight the representative heterotrophic microbes engineered for C1 fixation and in vitro tandem enzymatic systems for assimilating C1 sources to more complex molecules.

### Artificial chemoautotrophic systems

In recent years, there has been a notable advancement in engineering heterotrophic microorganisms for the biomass conversion of CO_2_ into biomass. Milo and coworkers [36] successfully enabled *E. coli* to generate all biomass carbon from CO_2_ trough the CBB cycle for the first time. Meanwhile, the energy generated from formate oxidation provided extra reducing power for the CBB cycle. Chemostat-based directed evolution was used to convert *E. coli* from an obligate heterotroph to a full autotroph [36]. Similarly, Mattanovich and coworkers [14] engineered *P. pastoris* to the autotroph by introducing the CBB cycle followed by adaptive laboratory evolution (ALE). Since the methanol oxidation of *P. pastoris* is localized in the cytoplasm, the entire CBB cycle in the peroxisomes was driven by the energy from the native methanol assimilation pathway [14]. However, introducing the CBB cycle into heterotrophs typically requires extra 9 molecules of adenosine triphosphate (ATP) and 4 molecules of nicotinamide adenine dinucleotide (phosphate) [NAD(P)H], which can cause extreme energy imbalance for the host strain and growth retardation [7]. Formate and methanol are used by microbes to produce NAD(P)H (Fig. 2D). A couple of enzymes are involved in this process, including formate dehydrogenase (FDH) [36], formaldehyde dehydrogenase (FaldDH), and alcohol oxidase 1 [14]. Additionally, ATP can be synthesized from NAD(P)H through phosphorylation [37].

Formate can be used directly as a carbon source. Several studies have focused on engineering microbes to utilize formate via the rGly pathway, a versatile route for C1 biotechnology [38–40]. In this metabolic pathway, formate is initially transformed into methylene-THF, which involves the catalytic action of formate-THF ligase, methenyl-THF cyclohydrolase, and methylene-THF dehydrogenase. Then, methylene-THF, CO_2_, and ammonia are condensed to glycine by the GCS. Finally, glycine is condensed with another methylene-THF to form serine, which is further converted to pyruvate [41]. This pathway fixes one CO_2_ molecule from formate to serine. An alternate pathway for CO_2_ fixation in heterotrophic microbes involves the use of a carbon capture enzyme called FDH, which facilitates the reversible reaction between CO_2_ and formate [42,43]. NAD-independent FDH has high affinity for CO_2_ but is sensitive to oxygen [44], while NAD-dependent FDH has lower activity toward CO_2_ but is not affected by oxygen [45]. Lee and coworkers [46] engineered *E. coli* to utilize formic acid and CO_2_ as the sole carbon source by integrating the reconstructed THF cycle and the reverse glycine cleavage reaction, optimizing metabolic fluxes, and adjusting cytochrome bo3 and bd-I ubiquinol oxidase levels. The genetically modified *E. coli* achieved an optical density at 600 nm of 7.38 within 450 h [46]. In this work, formate was also oxidized to CO_2_ through the expression of heterologous *Candida boidinii FDH* and *Arabidopsis thaliana fdh*_mut_ for redox regeneration [46]. Alternatively, Bar-Even and coworkers [24] fueled the rGly pathway by the oxidation of methanol to formate and enabled *E. coli* to grow on formate alone. The energy module operates as follows: Methanol is assimilated through the activity of methanol dehydrogenase (MDH), where formaldehyde is oxidized to formate through native glutathione system activity [24]. After that, Lindner and colleagues [47] demonstrated the potential of utilizing the rGly pathway for bioproduction by showing that *E. coli* can support a sustainable, formate-based bioeconomy. They achieved this by engineering lactate production from formate and CO_2_ through ALE and optimization of the rGly pathway [47]. Except *E. coli,* rGly has also been proved to work in other heterotrophic microbes such as *S. cerevisiae* [48], *Cupriavidus necator* [39], *Desulfovibrio desulfuricans* [9], and *Komagataella phaffii* [49], which paves the way toward metabolic engineering of formate and CO_2_ utilization for the production of proteins, biomass, or chemicals in heterotrophs.

XuMP pathway (Fig. 2A and B). Antoniewicz and coworkers [50] developed an *E. coli* strain that can synthesize all amino acids from methanol-derived carbon through ALE. In this study, methanol was first oxidized to formaldehyde by expressing the *Bacillus stearothermophilus* MDH gene and then assimilated through the RuMP cycle [50]. Unlike *P. pastoris* and *O. polymorpha*, *S. cerevisiae* does not possess a native methanol assimilation pathway [51,52]. To address this issue, Williams and coworkers [51] engineered *S. cerevisiae* to assimilate methanol through ALE. A system biology approach showed that a significant alteration in central carbon metabolism fluxes and gene expression took place, such as the truncation of an uncharacterized transcriptional regulator, Ygr067cp [51]. This discovery may provide insight into the fundamental understanding of methylotrophy in *S. cerevisiae*. Subsequently, Nielsen, Keasling, Chen, and Bai collaboratively engineered *S. cerevisiae* to grow in minimal medium supplemented with methanol by using a thermodynamic-based module circuit (TMC) strategy followed by ALE. This TMC strategy includes evaluating combinatorial methanol-utilizing pathways in yeast, a compartmentalization strategy that enhances methanol utilization, and a modularization strategy that enhances methanol utilization in methylotrophic *S. cerevisiae*. The growth was limited, and the maximum OD_600_ of 0.75 could be achieved after 144 h in a minimal liquid medium where 1% methanol was used as the sole carbon source [53]. In addition, a variety of studies have been reported on developing synthetic methylotrophy in *S. cerevisiae* [54–56]. In comparison, *Yarrowia lipolytica* and *O. polymorpha* with the native XuMP or RuMP pathway for methanol assimilation have been engineered to efficiently use methanol as the only carbon source to produce valued compounds [52,57,58].

In addition, CO_2_ can be electrocatalytically reduced to intermediates, such as methanol, CO, and formate [59,60], which are further consumed by microbes for valuable compound production [61]. Xia, Yu, and Zeng collaboratively develop a 2-step electrolysis process to reduce CO_2_ to CO and then a pure acetic acid. Then, they proposed a hybrid electro-biosystem that combined spatially separate CO_2_ electrolysis with yeast fermentation to efficiently convert CO_2_ to glucose at a high yield. Additionally, they genetically engineered *S. cerevisiae* by deleting specific hexokinase genes and overexpressing heterologous glucose-1-phosphatase, enabling the production of glucose from electrogenerated acetic acid in vitro. This platform also demonstrated the potential for easy expansion to produce other products, such as fatty acids, using CO_2_ as the carbon source [61]. These results highlight the exciting prospect of a renewable electricity-driven manufacturing industry [61]. Some of the remarkable progresses and emerging strategies in projecting electrobiochemical systems were shown in other reviews [62].

The abovementioned studies suggest that energy imbalance, oxygen sensitivity, and poor activities of rate-limiting enzymes of C1 fixation pathways (Table) might prevent us from developing efficient chemoautotrophic biological systems for C1 fixation. For example, the CO_2_ fixation efficiency of the CBB cycle is limited by the poor activity and selectivity of RubisCO and the imbalanced energy: 9 extra molecules of ATP and 4 molecules of NAD(P)H are consumed through the process [7]. Thus, the energy balance among the heterologous C1 assimilation pathway and the native endogenous pathway is crucial for constructing an efficient chemoautotrophic strain. The utilization of the ALE strategy is a prevalent approach for the purpose of biosystem self-regulation. However, its implementation can be time-consuming. For example, the CBB cycle in *E. coli* required approximately 200 days to evolve for CO_2_ assimilation [36], while *P. pastoris* took around 275 days [14]. Additionally, it took approximately 13 months for *S. cerevisiae* to evolve XuMP pathways for methanol assimilation [51]. Considering that the carbon fixation efficiency is also limited by the poor activities and oxygen sensitivity of key enzymes in the natural C1 assimilation pathways discussed so far (Table), designing artificial C1 fixation pathways to circumvent those limitations might be necessary for making the breakthrough in engineering heterotrophic microbes for C1 utilization [63].

**Table.** Key factors in C1 fixation pathways

Carbon species fixed	Pathways	Key enzymes	ATP equivalents	NAD(P)H equivalents	Oxygen tolerance	Theoretical carbon yield ^a^
CO_2_	The CBB cycle ^b^	RuBisCO [171,172]	9	4	Yes	50% (1 Ac-CoA/2 glycolate)
CO_2_	Reductive glycine pathway ^c^	Reductive glycine cleavage complex [173]Pyruvate-ferredoxin oxidoreductase [9]	2	4	Yes	50% (1 Ac-CoA/2 glycolate)
CO_2_	Wood–Ljungdahl pathway	FDH [174]CO dehydrogenase [175]Formylmethanofuran dehydrogenase [176]Pyruvate-ferredoxin oxidoreductase [9]	<1	4	No	100% (1 Ac-CoA)
CO_2_, bicarbonate	DC/HB cycle	4-Hydroxy butyryl-CoA dehydratase [177]Pyruvate-ferredoxin oxidoreductase [9]	5	4	No	100% (1 Ac-CoA)
Bicarbonate	3-HP bicycle ^d^	Malonyl-CoA reductase [178]	7	4	Yes	100% (1 Ac-CoA)
Bicarbonate	HP/HB cycle	4-Hydroxy butyryl-CoA dehydratase [177]	6	4	Yes	100% (1 Ac-CoA)
CO_2_	Reductive TCA cycle	2-Ketoglutarate synthase [67]ATP-citrate lyase [179]Pyruvate-ferredoxin oxidoreductase [9]	2	4	Yes	100% (1 Ac-CoA)
Formate	Reductive glycine pathway	Reductive glycine cleavage complex [173]	2	3	Yes	50% (1 Ac-CoA/2 glycolate)
Methanol	RuMP cycle or the CBB cycle or serine cycle or XuMP cycle	Alcohol oxidase [180]Methanol dehydrogenase [50]	–	–	Yes	50% (1 Ac-CoA/2 glycolate)
CO	Desulfurication-related pathways	CO dehydrogenase (CODH) [35]	–	–	Yes	–
CO_2_ (in vitro)	CETCH cycle ^d^	Crotonyl-CoA carboxylase/reductase [78,181]	1	4	Yes	100% (1 Ac-CoA)
CO_2_, bicarbonate (in vitro)	rGPS-MCG cyclePOAP cycle	Crotonyl-CoA carboxylase/reductase [66]Phosphoenolpyruvate carboxylase [66]Pyruvate synthase [74]	51	50.5	YesYes	100% (1 Ac-CoA)100% (1 Ac-CoA)
CO_2_ (in vitro)	ASAP	–	3	–	Yes	–
Formaldehyde (in vitro)	SACA pathway (in vitro/in vivo)	Glycolaldehyde synthase; acetyl-phosphate synthase [73]	–	–	No	100% (1 Ac-CoA)
GAPA pathway	Fructose 6-phosphate phosphoketolase [86]	–	–	No	–
CO_2_, bicarbonate (computation)	rCCC	Pyruvate carboxylase [182]Crotonyl-CoA carboxylase/reductase [66]	3	5	Yes	–
CO_2_ (computation)	2-HG-rTCA cycle	Isocitrate dehydrogenase [63]	2	3	Yes	–

^a^The carbon yield is calculated based on the production of acetyl-CoA.

^b^The CBB cycle initially produces glyceraldehyde-3-phosphate. Here, we assume that one molecule of glyceraldehyde-3-phosphate produces one molecule of acetyl-CoA, one molecule of CO_2_, and 2 molecules of NADH.

^c^The reductive glycine pathway and the 3-HP bicycle initially produce pyruvate. Here, we assume that one molecule of pyruvate produces one molecule of acetyl-CoA, one molecule of CO_2_, and one molecule of NADH. The CBB cycle originally produces glyceraldehyde-3-phosphate. Here, we assume that one molecule of glyceraldehyde-3-phosphate produces one molecule of acetyl-CoA, one molecule of CO_2_, and 2 molecules of NADH.

^d^The glyoxylate produced by the CETCH cycle is not adjusted to acetyl-CoA when calculating the energy and reducing equivalents.

### In vitro tandem enzymatic systems for C1 fixation

Constructing heterotrophic microbes for stable growth rates and high carbon yield with C1 sources is challenging due to inherent metabolic inefficiencies and the unfavorable cross-talk between product-forming and growth-sustaining reactions [64,65]. Establishing an in vitro tandem enzymatic system has several advantages and theoretically can improve C1 fixation efficiency. In the absence of competing pathways and metabolic burden or toxicity, the yield can approach the theoretical maximum much more closely than the in vivo systems. Moreover, the system’s simplicity makes it more feasible to optimize via modeling and enzyme concentration adjustments [66]. However, combining multiple enzymes tandemly for C1 fixation does not merely replicate the catalytic behavior of each enzyme. Several strategies must be considered to maintain the stability of metabolites and enzymatic activity under reaction conditions and balance the reaction rate of each step. For example, the self-replenish cycles are preferred to ensure that the intermediates are self-replenished and balanced for continuous metabolic flow [67,68]. Additionally, the enzymes in the cycle should be purifiable and have high activity, specificity, and stability under aerobic condition [68]. The oxidoreductases’ cofactors should be dynamically regulated with balanced consumption and regeneration rate. Last but not least, all enzymatic steps should be thermodynamically favorable, and the theoretical carbon yield should approach 100% [69,70].

**Fig. 3.**
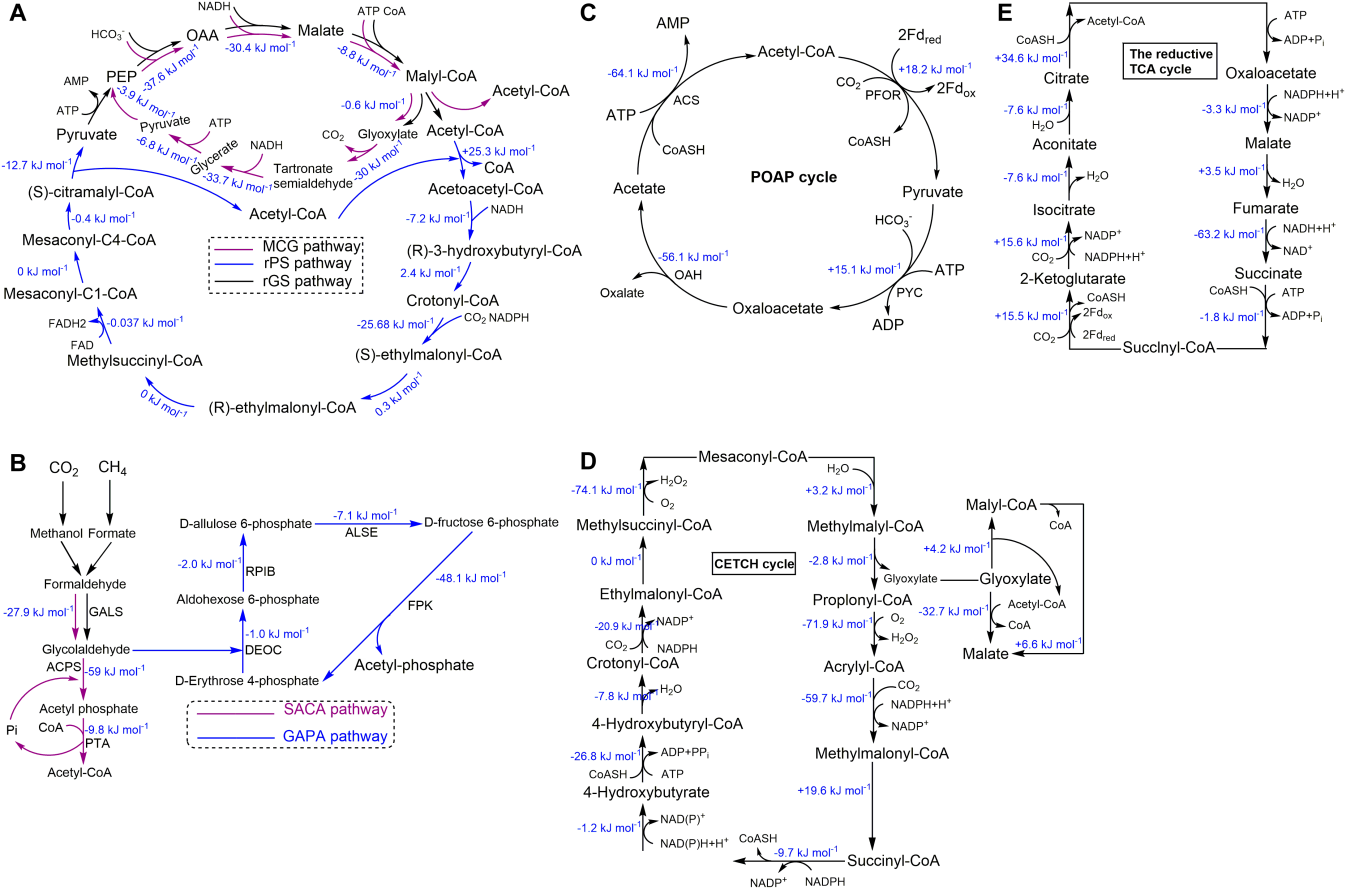
Existing artificially designed CO_2_ fixation pathways. (A) rGPS-MCG pathway [66]. The rGPS cycle consists of the rGS pathway (black) and the rPS pathway (blue). The MCG pathway (purple) consists of the rGS and glycerate pathways. (B) SACA and GAPA pathway for novel and carbon-conserving formaldehyde (FALD) assimilation pathways [73,86]. (C) POAP cycle, a minimized synthetic CO_2_ cycle [74]. (D) CETCH cycle. This cycle is a synthetic CO_2_ fixation pathway verified in vitro [78]. (E) ASAP pathway. The ASAP pathway fixes 1 mol of CO_2_ by chemical hydrogenation under anabolic condition in vitro. The ASAP pathway fixes 1 mol of CO_2_ by chemical hydrogenation under anabolic condition in vitro [85]. The changes in the Gibbs energy calculated by eQuilibrator [183,184] are shown in blue.

In recent years, many artificial C1 fixation pathways have been modified based on the natural C1 pathways (Fig. 3). Constructing artificial C1 fixation pathways for synthesizing acetyl-CoA from one-carbon sources is a vital task, as acetyl-CoA serves as a crucial metabolite for life on Earth and a primary precursor for diverse industrial chemicals and natural products biosynthesis [71,72]. Liao and coworkers [66] built an enzymatic network consisting of a synthetic reductive glyoxylate, pyruvate synthesis (rGPS) cycle, and the malyl-CoA-glycerate (MCG) pathway to convert a C3 metabolite into 2 acetyl-CoA while fixing one CO_2_ or assimilate glyoxylate to acetyl-CoA without the overall carbon loss (Fig. 3A). By utilizing an opto-sensing and control module for dynamic cofactor regeneration, they achieved continuous operation for up to 6 h with a CO_2_ fixation rate comparable to or exceeding those of typical photosynthetic or lithoautotrophic organisms [66]. After that, they repurposed glycolaldehyde synthase and acetyl-phosphate synthase to engineer a synthetic acetyl-CoA (SACA) pathway (Fig. 3B) that was the most efficient, oxygen-insensitive, ATP-independent, and carbon-neutral approach for synthesizing acetyl-CoA [73]. Meanwhile, Li and coworkers [74] developed a compact synthetic CO_2_ fixation cycle (POAP) using pyruvate carboxylase, oxaloacetate acetylhydrolase, acetate-CoA ligase, and pyruvate synthase to work as a model system to study the CO_2_ fixation in the earliest organisms on earth (Fig. 3C). The purpose of fixing CO_2_ in vitro is to synthesize valuable compounds from C1. Zeng and coworkers [75,76] developed an ATP- and NAD(P)H-free chemoenzymatic systems to fix atmospheric CO_2_ and NH_3_ into amino acids. This systems involved a modified GCS, where the NAD(P)H-dependent dihydrolipoyl dehydrogenase was substituted by biocompatible chemical reduction of protein H using dithiothreitol [75,76]. Subsequently, Zhu and coworkers [77] developed a tandem enzymatic system consisting of rGly pathway integrated with an electrocatalytic system to synthesize glycine from CO_2_ and NH_3_ directly. These results create an avenue for the direct production of amino acids and their derivatives from CO_2_.

Natural C1 fixation cycles, such as the CBB cycle, rGly, and rTCA, suffer from carbon loss and thus low carbon fixation rate (Table). Therefore, developing artificial C1 fixation pathways is necessary to mitigate these issues. Efforts toward creating de novo artificial pathways with superior yields and activity than the natural ones remain a priority. Erb and coworkers constructed the crotonyl-CoA/ethylmalonyl-CoA/hydroxybutyryl-CoA (CETCH) synthetic pathway (Fig. 3D), achieving continuous conversion of CO_2_ into malate at a rate of 5 nmol per minute per milligram of protein [78], which outperforms chemical processes and rivals native CO_2_-fixing pathways in vitro. The CETCH cycle was designed and constructed based on the freely available databases, such as KEGG [79] and MetaCyc [80]. To power the CETCH cycle, Baret and Erb collaboratively developed an artificial chloroplast by encapsulating photosynthetic membranes in cell-sized droplets and used the energy generated from the light reaction of photosynthesis to drive the CETCH cycles [81]. Most recently, Burnetti and Ratcliff collaboratively engineered *S. cerevisiae* into a facultative phototroph by introducing a rhodopsin protein from *Ustilago maydis* into the vacuole, enabling light-driven proton pumping into the vacuolar compartment without consuming ATP. The findings demonstrated that the yeast engineered with rhodopsins exhibit a selective growth advantage under green light, displaying faster growth rates than their nonphototrophic ancestor and rhodopsin-expressing yeast grown in the dark [82]. To convert CO_2_ into value-added compounds continuously, Erb and coworkers [83] created a modular platform for continuously converting CO_2_ to acetyl- and malonyl-CoA that were converted into monoterpenes, sesquiterpenes, and polyketides via the use of varied terpene and polyketide synthases. This work paves the way for engineering synthetic routes for generating complex chemicals from CO_2_ in the future. To improve the carbon fixation efficiency and reduce the energy consumption in the CBB cycle and CETCH cycle, Erb and coworkers have made further improvements by developing a carboxylation module consisting of a new-to-nature glycolyl-CoA carboxylase (GCC), an engineered glycolyl-CoA synthetase (GCS), and an engineered tartronyl-CoA reductase (TCR) to convert glycolate into glycerate. They predict that the integration of carboxylation module with photorespiration, ethylene glycol conversion, and CETCH cycle can increase the carbon efficiency of all these processes by up to 150% and reduce their theoretical energy demand [84]. Additionally, Ma and coworkers constructed an artificial starch anabolic pathway (ASAP) in a cell-free system, consisting of main set reactions predicted by using MetaCyc and ATLAS databases [85] (Fig. 3E). The ASAP pathway was developed via modular assembly and substitution, with optimized protein engineering of 3 bottleneck-related enzymes (formolase, fructose-bisphosphatase, and adenosine diphosphate–glucose pyrophosphorylase) [12]. The finalized ASAP was driven by hydrogen and can convert CO_2_ to starch at a rate of 22 nmol of CO_2_ per minute per milligram of total catalyst, which is approximately 8.5 times higher than the rate of starch synthesis in maize (Table). Apart from the novel CO_2_ fixation pathways, C1 assimilation pathways could be designed based on the artificial aldolase (ALS) reactions systematically. Ma and coworkers used comb-flux balance analysis (FBA) algorithm to design 8 carbon-conserving formaldehyde (FALD) assimilation pathways based on an extended metabolic network with nonnatural aldol reactions using the comb-FBA algorithm. In vitro, a new FALD assimilation pathway utilizing allose 6-phosphate, termed the glycolaldehyde-allose 6-phosphate assimilation (GAPA) pathway, was developed with a high carbon yield of 94% (Fig. 1J) [86].

In summary, artificial C1 fixation pathways are generally designed based on the simulations and predictions by using KEGG [79], MetaCyc [80], ATLAS [85], and novoPathFinder databases [87]. Combinatorial algorithm and parsimonious flux balance analysis (comb-FBA) [65] and labeling flux analysis [88–90] are used to determine the thermodynamic efficiencies of C1 fixation processes. In addition, a strategy of modular assembly and substitution is used to address the limitations induced by the unpredictable and undesired interactions among enzymes from disparate biochemical contexts in computationally designed pathways [12]. Despite the successful proof of concept of various cell-free enzymatic systems, more efforts are needed to verify newly designed pathways and address the problems such as enzymatic stability and dynamic control for industrial-scale applications.

## Chemo-Bio Hybride Systems for CO_2_ Fixation

Except for engineered biological systems used for CO_2_ fixation, artificial photosynthesis catalyzed by photocatalysis, chemical catalysis, and electrocatalysis provides an alternative for CO_2_ utilization [7]. Photocatalysis and electrocatalysis allow for flexible conversion between light (electrical) energy and chemical energy, representing a favorable means for sustainable green chemistry applications in CO_2_ utilization. However, electrocatalytic and photocatalytic CO_2_ reductions are thermodynamically uphill and require substantial energy input for breaking the C=O bond. Even worse, CO_2_ reduction has poor selectively and can yield various reduction products, including CO, formic acid, methane, and ethylene, by using different electrocatalysts and photocatalysts and is performed under different conditions. So, it is even harder to synthesize high-value chemicals via direct CO_2_ electrocatalysis and photocatalysis [91–93]. Considering that biocatalysts can selectively and efficiently synthesize value-added chemicals from C1 feedstocks (CO_2_, formaldehyde, formate, and methanol), there is a growing interest to construct electrical-bio and photoelectrical-bio systems for synthesizing value-added compounds from CO_2_ by combining the synthetic power from both disciplines. By combining the advantages of electrocatalysis (photocatalysis) and biocatalysis, chem-bio hybrid systems aim to produce value-added chemicals from CO_2_ with high conversion efficiency and selectivity [94,95]. In this section, we will highlight one-pot chemo-bio hybrid systems for CO_2_ fixation.

### Bioelectrocatalytic systems for CO_2_ fixation

Bioelectrocatalysis is an interdisciplinary research field that combines electrocatalysis and biocatlaysis by using enzymes or whole cell as catalysts for redox reactions at an electrode [96]. One of its applications is bioelectrocatalytic CO_2_ reduction to C1 chemicals (formate, formaldehyde, methanol, and methane) by either enzymes derived from carbon assimilation pathways or electrocatalysts [3]. These C1 chemicals can be further converted into various fuels and commodity chemicals through enzymatic reactions in vitro or in vivo [97]. Bioelectrocatalytic CO_2_ reduction has several advantages over conventional enzymatic CO_2_ reduction: It can use renewable electricity as electron source, it can achieve high-energy conversion efficiency, and it can exploit the selectivity and specificity of enzymes or whole cells. Therefore, bioelectrocatalytic CO_2_ reduction is a promising strategy for replacing petroleum-based feedstocks with sustainable and environmentally friendly alternatives.

#### Electroenzymatic systems

As discussed before, electrochemical CO_2_ reduction is limited in requiring a high overpotential, leading to reduced selectivity, catalytic yield, and specificity [98]. Bioelectrocatalytic reduction of CO_2_ using oxidoreductases is one of the effective solutions. Enzymes can effectively catalyze CO_2_ reduction at or near its thermodynamic potentials, thus reducing the electrochemical overpotential required to drive the reaction [99].

**Fig. 4.**
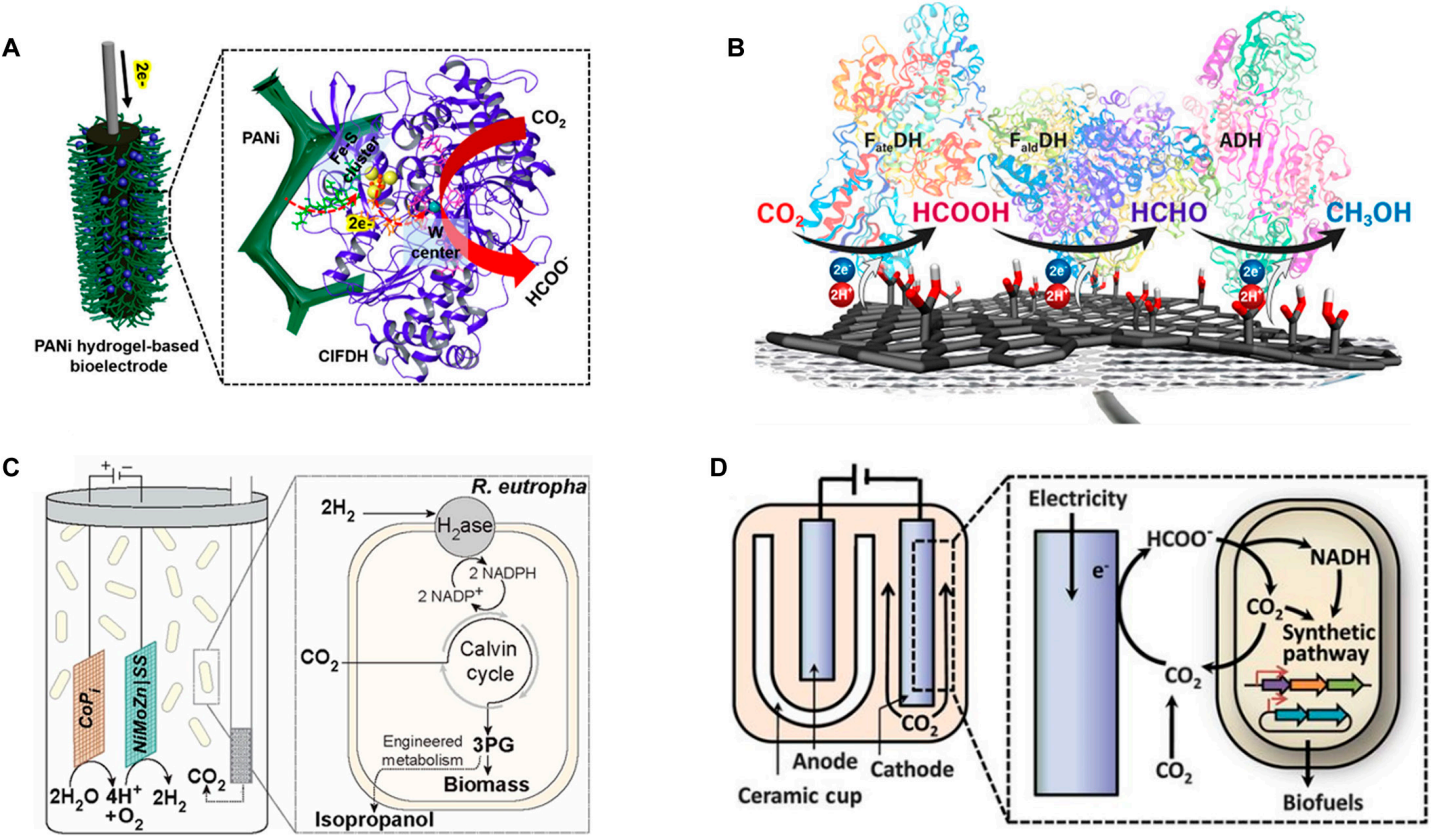
Schematic illustration of electrical-bio system for CO_2_ fixation. (A) Direct electron transfer from the conductive PANi hydrogel electrode to immobilized *Cl*FDH for electroenzymatic CO_2_ reduction to formate [104]. (B) Direct electron transfer from the functionalized graphene to the immobilized tandem enzymatic cascade for CO_2_ reduction to methanol [105]. (C) Culturing of *C. necator* in bioelectrochemical cell with CoPi anode and NiMoZn/SS cathode to convert H_2_ and CO_2_ into 3PG that is subsequently converted into biomass or isopropanol in engineered Re2133-pEG12 [118]. (D) Electrochemical reduction of CO_2_ to formate that is converted to isobutanes and 3MB by the engineered *R. eutrophic* [120].

Since the active sites of oxidoreductases are usually placed deep within the enzymes, a limited number of them can obtain the electrons from the cathodes directly without any modifications through the direct electron transfer mechanism (DET) [96]. Instead, mediators are needed as electron shuttles between the electrode and the active sites of enzymes. NADH and NADPH are the most common electron carries for oxidoreductases catalyzing CO_2_ reduction [100]. In 1999, Obert and Dave [101] collaboratively illustrated CO_2_ reduction to methanol using FDH, formaldehyde dehydrogenase (FaldDH), and alcohol dehydrogenase (ADH) by costing 3 NADH molecules. This breakthrough has facilitated the bio-catalytic production of molecules possessing high-energy content, accompanied by an efficient combustion/energy dissipation mechanism. However, the yield of formic acid was low simply because the reverse formic acid oxidation rate is much faster than the forward reduction rate of CO_2_ [102]. Moreover, NADH is expensive and unstable cofactor. Although its enzymatic regeneration systems are relatively matured, the accumulation of site products complicates the downstream separation processes, which further increases the cost of any large-scale applications [102]. Therefore, activating oxidoreductases through the direct electron transfer strategies is more desirable, which can be achieved by integrating the enzyme on the functionalized electrodes after taking electrodes’ topology, porosity, and surface chemistry (hydrophilicity, surface charge, and functional moieties) into consideration [103]. Lee and Park [104] collaboratively developed an electro-enzymatic CO_2_ reduction system consisting of a conductive polyaniline (PANi) hydrogel conjugated with W-containing FDH from *Clostridium ljundahlii* (*Cl*FDH), in which *CI*FDH was regenerated through the direct electron mechanism and a 92.7% Faradaic efficiency (FE) can be achieved (Fig. 4A). Later, Seelajaroen and coworkers [105] explored NADH-free electroreduction of CO_2_ to methanol catalyzed by the hydrogenases (FDH, FaldDH, and ADH)-graphene hybrid (Fig. 4B). This nanobiocatalyst reduced CO_2_ to methanol at high current densities without producing other soluble by-products for at least 20 h of operation.

#### Electromicrobial systems

Microbial electrosynthesis (MES) is a technology that uses microorganisms as biocatalysts to reduce CO_2_ with the electrons from cathodes as the reducing equivalents [106,107]. This technology relies on the inward electron transfer mechanisms that enable microorganisms to synthesize value-added chemicals. However, not all microorganisms can directly uptake the electrons from electrodes via extracellular electron transfer (EET) mechanisms. For example, many acetogens such as *Sporomusa* species and *Moorella thermoacetica* can perform EET [108,109], but other non-electroactive bacteria such as *C. necator* and *E. coli* cannot. To overcome this limitation, non-electroactive bacteria have been adopted to indirectly uptake electrons from cathodes via electron shuttles [110,111], like methyl viologen (MV) [112], quinone analogs [113], and neutral red (NR) [114,115], which increases their intracellular reducing equivalents, including NADH that facilitates CO_2_ reduction. Electron shuttles have been widely explored to enable the synthesis of various chemicals by different microorganisms in MES. Among them, NR is widely used due to its comparable standard reduction potential (−525 mV versus Ag/AgCl) to that of NADH/NAD^+^ (−520 mV versus Ag/AgCl) [116], which makes it able to interact with metabolic steps in turn to improve utilization of reducing equivalents.

Besides electron shuttles, hydrogen and formate produced by inorganic catalysts (such as indium and cobalt–phosphorus) can also work as electron carriers for CO_2_ fixation. For instance, *C. necator* can use these electron carriers to produce biofuels such as butanol and isobutyl alcohol [117]. Nocera and coworkers [118] developed a water-splitting biosynthesis system, using a cobalt phosphate catalyst as an anode and a cobalt–phosphorus alloy cathode for oxygen reduction and hydrogen evolution, respectively (Fig. 4C). *C. necator* oxidized H_2_ in the cathodic chamber, producing NADPH and ATP as reducing equivalents to enable the conversion of CO_2_ to poly-β-hydroxybutyrate (PHB). However, at ambient pressure and room temperature in water, H_2_ exhibits low solubility (0.00016 g/100 g H_2_O) when compared to CO_2_ (0.169 g CO_2_/100 g H_2_O) [119], which limits its mass transfer and electron delivery rates. To overcome this limitation, Liao and coworkers [120,121] developed a system to generate formate from CO_2_ electrocatalytically by using an indium cathode (Fig. 4D). Formate was then converted to isobutyl alcohol and 3-methyl-1-butanol by the engineered *C. necator* [120,121].

Optimizing the electrode surface is also crucial for supporting fast electron transfer rates. The morphology and chemistry of electrodes affect the efficiency of MES in terms of biofilm formation and the electron transfer mechanisms between microbes and electrodes. Electrodes’ materials selections and surface modifications have been done to achieve higher MES efficiency by improving its (a) biocompatibility [122], (b) electrochemical surface area [123], (c) electron transfer rates [124], (d) conductivity [125], and (e) mass transfer efficiency between substrates and products [126]. The interaction between the electroactive microbes and the electrodes can also be used to investigate the electron communications on the nanometer scales.

### Photo-bio hybrid systems for CO_2_ fixation

Photosynthesis is a natural process evolved by cyanobacteria, algae, and green plants to capture solar energy while oxidizing water to oxygen and drive the conversion of CO_2_ into biomass and secondary metabolites [127]. This process forms the basis for all existing life today [128]. However, biology suffers from relatively poor solar energy conversion efficiencies, as conventional crops only have a 0.5 to 1% solar-to-biomass conversion efficiency [129]. Artificial photosynthesis integrates biocatalysts with synthetic materials systematically and realizes the solar-to-chemical conversion comparable to or even better than that of the natural one [130]. In these hybrid systems, enzymes or microbes catalyze selective chemical transformations and synthetic materials are scaffolds for immobilizing biocatalysts and chemical catalysts for light absorption, charge transfer, and chemical reactions. The development of photo-bio hybrid systems typically entails selecting an appropriate enzyme or microorganism for chemical synthesis, employing a semiconductor nanomaterial as photosensitizer, evaluating the synergistic effects of the inorganic–biological hybrid systems, and investigating the energy transduction mechanisms at newly formed biotic–abiotic interfaces.

#### Photoenzymatic systems

**Fig. 5.**
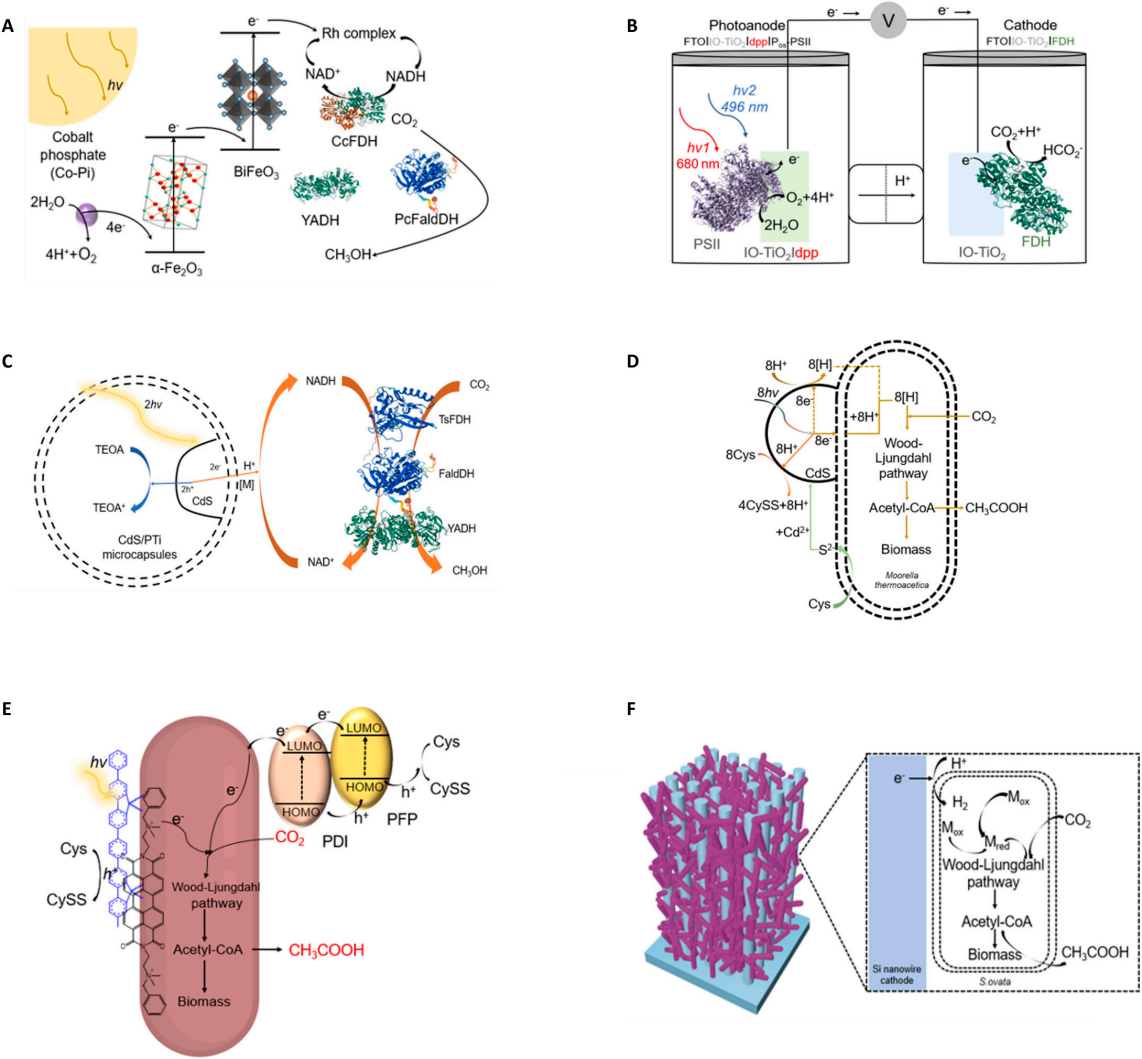
Schematic illustration of photo-bio hybrid system for CO_2_ fixation. (A) Solar-assisted reduction of CO_2_ to methanol by tandem enzymatic cascade [133]. (B) Semi-artificial tandem PEC cell couples CO_2_ reduction to water oxidation through photosynthesis. A blend of POs and PSII adsorbed on a dpp-sensitized photoanode (IO-TiO_2_|dpp|POs-PSII) is wired to an IO-TiO_2_|FDH cathode [141]. (C) CdS QDs on the capsule wall absorb visible light to generate electrons and holes. The electrons are transferred to the outer surface of the capsule wall through the heterostructure of CdS and amorphous titania and then transduced to methanol through reduced NADH by formate dehydrogenase from *C. boidinii* (*Cb*FDH), FaldDH from *Pseudomonas* sp. (*P.sp*FaldDH), and yeast alcohol dehydrogenase (YADH) [142]. (D) A hybrid system of *M. thermoacetica* and CdS nanoparticles converts CO_2_ to acetic acid through photosynthesis, by bioprecipitation (loading) of CdS nanoparticles during cell growth [149]. (E) An organic semiconductor–bacteria biohybrid photosynthetic system is presented, which efficiently reduces CO_2_ to produce acetic acid through the nonphotosynthetic bacteria *M. thermoacetica* [153]. (F) A close-packed nanowire–bacteria hybrid system (left) is schematically depicted with its reaction pathway (right) [154].

Photoelectrochemical tandem cells that combine semiconductor’s light-harvesting capability with enzymatic CO_2_ fixation process show immense potential for selectively converting CO_2_ to value-added chemicals efficiently with low carbon footprint and a low energy input [130–137]. Creating a biocompatible interface and ensuring the efficient electron transfer from photocatalysts to biocatalysts is essential to realize the synergic effects between photocatalysis and biocatalysis [138–140]. For instance, Lee and coworkers [133] developed a photoenzymatic hybrid system that combined an enzymatic cascade with a Z-scheme architecture consisting of a Co-Pi/α-Fe_2_O_3_ photoanode and a BiFeO_3_ photocathode (Fig. 5A). By using this device, the electrons generated from the water oxidation on the photoanode were used to regenerate NADH that provides the reducing power for CO_2_ to methanol conversion catalyzed by *Cc*FDH-*Pc*FaldDH-YADH enzymatic cascade in the photocathode chamber. The Z-scheme systems realized 80% NADH regeneration efficiency under 0.8 V bias by using water as the electron donor with limited side product formations.

Photoelectrochemical (PEC) tandem cells have the potential to enable the electron transfer from the photosynthetic light reactions to other CO_2_ fixation pathways rather than the CBB cycle. Reisner and coworkers [141] introduced a semi-artificial PEC tandem cell that coupled photosystem II (PSII) to FDH for formate formation from CO_2_ using water as the electron donor and energy from the light (Fig. 5B). The conducting wire facilitates directional transfer of reducing equivalents from photoelectrodes to biocatalysts, while a flat-sheet semipermeable membrane isolates photocatalytic oxidation and biocatalytic CO_2_ reduction processes to maintain the catalytic activity. This semi-artificial architecture realized the complementary light absorption and coupling of unnatural redox partners together, which is challenging in vivo. Facile and controllable methods need to be developed to optimize the efficiency of photocatalysis and biocatalysis, respectively, in PEC tandem cells [141,142].

Besides PEC tandem cells, light-sensitive nanoparticles have also been coupled with various oxidoreductases for different chemical transformations. The major works in this area focused on hybridizing nanoparticles, especially TiO_2_, with various oxidoreductases to reduce CO_2_ to CO, formic acid, formaldehyde, methyl alcohol, and methane [143–146]. In order to reduce the damage of enzymes due to photogenerated holes and reactive oxygen species (ROS) from semiconductors, Jiang and Shi [142] collaboratively designed the artificial thylakoids by decorating protamine–titania (PTi) microcapsules’ inner walls with cadmium sulfide (CdS) quantum dots to efficiently regenerate NADH, which drives the tandem enzymatic reactions catalyzed by FDH, FaldDH, and ADHs for CO_2_ reduction (Fig. 5C). By using this system, CO_2_ can be transformed into formate with a quantum yield of 0.66 ± 0.13%, similar to the annual average observed in natural plants (approximately 0.2 to 1.6%) [130].

#### Photomicrobial systems

The photomicrobial system combines photocatalytic material, such as semiconductors, electrodes, polymers, and dyes, with microbes to realize CO_2_ fixation at broader spectrum wavelength and higher solar energy conversion efficiency [147]. The self-replication, self-optimization, and self-healing capabilities of microbes offer potential stability and scalability advantages for cell-based systems [130].

In semiconductor–microbial hybrid systems, semiconductor materials commonly distributed in medium, immobilized on the cell membrane, or inside the cell. Light-induced excitation of a semiconductor material generates electron-hole pairs that are harnessed by tandem enzymatic systems for chemical synthesis. When the semiconductor is extracellular, it needs a medicator to shuttle the electrons. Zhong and coworkers [148] designed a single-strain system for CO_2_ to formic acid reduction using MV as the mediator. However, the poor biocompatibility of MV led to cell death after the reaction [112]. Exclusion of mediators may be feasible by positioning semiconductors on the cell membrane, enabling photogenerated electrons to enter the cell membrane and engage in the intracellular enzymatic reactions directly. For instance, Yang and coworkers [149] deposited CdS nanoparticles onto a nonphotosynthetic *M. thermoacetica* surface (Fig. 5D). The harvested energy stimulated cellular metabolism, leading to excessive production of acetic acid. Guo and Joshi [150] collaboratively developed a biocompatible polyphenol-based assembly method, utilizing light-harvesting indium phosphide nanoparticles (InP) attached to the surface of *S. cerevisiae* for NADPH regeneration under illumination, culminating in shikimic acid production. Unlike CdS, the gold nanoclusters (Au NCs) with smaller sizes and better compatibility can cross the cell membrane without killing the microbes. The direct interaction of Au NCs with enzymes improved charge transfer rate and achieved a 33% increase in quantum efficiency for continuous CO_2_ fixation [95]. Except for Au NCs, biocompatible InP quantum dots (QDs) were also located inside the *E. coli* to drive the conversion of CO_2_ to acetate. The quantum efficiency (QE) of photosynthesis products can achieve 6 to 8%, resulting in a total productivity of approximately 0.89 mmol L^−1^ h^−1^. Genetically modified *E. coli* can subsequently convert acetate into amorphadiene [151].

Dye–polymer conjugates can work as photosensitizers to generate electrons under illumination and generally hybridize with the microbes through electrostatic force, van der Waals force, and physical action [152]. Wang and coworkers [153] coated *E. coli* with organic photosensitizers perylene diimide derivatives and poly(fluorene-co-phenylene) to form a polymer layer with high hole/electron separation efficiency and drive CO_2_ into acetic acid via the Wood–Ljundahl pathway (Fig. 5E). Compared with semiconductors, dye–polymer conjugates have fewer applications in artificial photosynthesis mainly because dyes are weaker reducing agent prone to photobleaching. Moreover, the energy bands of polymeric photosensitizers are hard to control since they are mainly regulated by functional group modifications and precise polymerization. Finally, the dye/polymer photosensitizer exhibits low biocompatibility and is environmentally hazardous [152].

In the electrode–microbial hybrid systems, photoelectrodes serve as sources of electrons, H_2_, or redox mediators that are transferred into the cell and participate in chemical synthesis. The electrode–microbial hybrid systems can be finely tuned for different purposes. A representative study is that Yang and coworkers [132] constructed a self-sufficient, solar-driven system with silicon (Si) and titanium dioxide (TiO_2_) nanowire arrays as a light-capturing “Z-scheme” and *Sporomusa ovata* as the biocatalysts to fix CO_2_ and generate acetate for up to 200 h under simulated sunlight, achieving energy conversion efficiencies of up to 0.38%. Limited interactions between microorganisms and electrodes resulted in suboptimal CO_2_ reduction efficiency and increased overpotential. To enhance the performance, they tuned the electrolyte pH for better buffering and constructed a compact nanowire-based cathode integrated with *S. ovata* to achieve efficient electroreduction of CO_2_ at a high applied potential (~−1.2 V versus standard hydrogen electrode) and a current density of 0.65 mA cm^−2^ (Fig. 5F) [154]. Moreover, the system achieved solar-driven CO_2_ fixation with a solar-to-acetate efficiency of approximately 3.6% over a period of 1 week. The low charge-transfer efficiency between a photoelectrode and bacteria limits the CO_2_ conversion efficiency in electrode–microbial hybrid systems. Further investigations should be conducted to enhance the chemical stability of microorganisms, biocompatibility of the electrodes, and electrical conductivity of their interfaces.

## Conclusions

Transforming heterotrophs into autotrophic organisms to use C1 compounds for self-sustaining metabolism is still challenging. It is difficult to anticipate the most effective C1 assimilation pathways in various heterotrophs due to the distinct metal chaperones, favorable redox environments, and membrane systems necessary for ATP coupling in differing pathways (Table) [155]. Moreover, the kinetics of each enzyme in a heterologous pathway also varies in hosts with different overall standard redox potential and intermediate concentrations. The efficiency of C1 fixation process is also primarily affected by the cultivation conditions and the targeted products. To put all these factors into considerations, comb-FBA [65] and labeling flux analysis [88–90] can be used to determine the thermodynamic efficiencies of C1 fixation processes. In addition, to enhance the adaptation of C1 fixation pathways, it is essential to rewire endogenous metabolic processes and further optimize novel synthetic routes in vivo or in vitro through advanced computational simulation tools [156,157]. Relying solely on established databases such as KEGG [79], MetaCyc [80], ATLAS [85], and novoPathFinder databases [87] may not be sufficient.

In vitro tandem enzymatic systems for C1 fixation have some limitations, such as the requirement of purified enzymes and well-controlled reaction conditions. It may encounter more complex issues in handling large-scale reactions and producing products at a commercially viable rate limited by poor enzymatic selectivity and the external supply of expensive cofactors. Using cell-free systems to incorporate cellular components can provide a more biomimetic environment for enzymatic reactions and improve their activity and specificity [158]. Designing artificial cofactors such as NADH and NADPH replacements and corresponding regeneration systems is an important strategy to reduce the cost of in vitro tandem enzymatic systems [159]. Meanwhile, developing machine learning or simulation model to precisely predict activity and selectivity of enzymes has the great potential to discover and engineer advanced enzymes more efficiently [160–163]. Additionally, using advanced high-throughput technology systems, such as droplet microfluidics [164–166], fluorescence-activated cell sorting (FACS)-based screening [167], microtiter plate-based screening [167], mass spectrometry (MS)-based screening [167], digital imaging [167], and automated facilities [168], can further shorten the research and development cycle by screening and optimization of reaction conditions. Meanwhile, in order to enhance assay development, sample preparation, variant measurements, and data analysis, the integration of novel technologies and interaction between different advanced techniques like automation and MS-based screening will be crucial [167].

An efficient energy module is crucial to successfully drive the artificial biological systems for C1 utilization. This review discusses three common energy supplies in artificial biological systems: chemical-to-chemical energy conversion, electrical-to-chemical energy conversion, and photo-to-chemical conversion. Introducing an energy module that can provide sufficient energy while minimizing carbon loss is essential for C1 utilization. For natural Aox-dependent methylotrophs like *P. pastoris*, the formaldehyde dissimilation pathway is vital for NADH production, but it results in over 40% carbon loss [169]. Reprogramming methanol oxidation pathway or constructing NADH regeneration systems may be alternative methods to resolve it [53,66,170]. The development of photosensitized microorganisms for efficient CO_2_ fixation represents a novel research avenue, establishing a new interface between nonphotosynthetic microbes and semiconductors through the confirmation of light-induced intracellular charge transfer, allowing for photoreduction of CO_2_. However, electron transfer without mediators remains challenging, and the oriented electron transfer between photosensitizers and the targeted biological charge transport chain is barely possible. Thus, further research on material science and molecular biology is needed to elucidate how microbes take up external electrons and possible abiotic electron transfer pathways in the hybrid systems. Finally, chemo-bio hybrids are hard to be scaled up due to their poor compatibility and difficulties in constructing self-assembly and disassembly systems. Designing and engineering self-regeneration and biocompatible living materials with high electroconductive efficiency will be a riveting field in the future.

In conclusion, developing an artificial biological system that can utilize C1 sources is an immensely impactful research endeavor due to their abundant sources, potential sustainable and carbon-neutral production, and lack of conflict with food security [53]. With recent advances in efficient in vivo gene diversification strategies, more biocompatible materials and electrical devices, more advanced high-throughput screening and automation platforms, and improved computational modeling and machine learning tools, developing artificial biological systems for C1 utilization is entering a new era to reveal the secrets of C1 fixation mechanism and engineer useful biological systems with a plethora of applications in the pharmaceutical, agriculture, and food industries.
